# Comprehensive Analysis and Leveraging Online Innovations to Improve HIV and STI Prevention and Treatment Services in Major Cities of Thailand

**DOI:** 10.1007/s10461-025-04714-x

**Published:** 2025-04-24

**Authors:** Panyaphon Phiphatkunarnon, Nittaya Phanuphak, Rena Janamnuaysook, Do Tran, Boon-Leong Neo, Worawit Tepsan

**Affiliations:** 1 International College of Digital Innovation, Chiang Mai University, 239 Nimmanhaemin Road, Suthep, Muang Chiang Mai, 50200 Thailand; 2 Institute of HIV Research and Innovation, Bangkok, Thailand; 3 Gilead Sciences Inc, Foster City, CA USA; 4 Gilead Sciences Inc, Singapore, Singapore; 5 Love Foundation, Chiang Mai, Thailand

**Keywords:** Digital health platforms, HIV prevention, mHealth, Public health innovation, STI service and prevention

## Abstract

Despite a 50% reduction in new HIV cases since 2010, HIV and sexually transmitted infections (STIs) remain pressing public health challenges in Thailand, particularly among marginalized and high-risk populations. This study examined 8,865 bookings made between October 1, 2023, and September 30, 2024, through Love2Test.org (L2T), a mobile platform launched in December 2020 to provide accessible sexual health services, including HIV testing and STI screening. Applying descriptive analysis, service utilization analysis, and K-means clustering, the study uncovered booking patterns, user demographics, and behavioral trends. L2T attracted a diverse user base, with 32.15% aged 15–24, 47.06% aged 25–34, 2.6% identifying as transgender women, and 16.59% as non-binary individuals, underscoring its inclusivity. HIV testing (38%) and STI screening (28%) were the most requested services, while Bangkok accounted for 74.5% of bookings, highlighting geographic disparities in access. Cluster analysis identified three user groups: older males seeking HIV tests, younger males and middle-aged non-binary individuals utilizing PrEP and STI screening, and a mixed group accessing both services. With 158,639 visitors and 8,865 service bookings, resulting in a visit-to-booking rate of 5.59%, L2T has proven instrumental in delivering confidential, targeted care to vulnerable groups. By addressing service gaps and promoting HIV testing, PrEP education, and STI prevention, L2T strengthens public health efforts. Expanding its outreach and improving service availability beyond Bangkok present opportunities to further reduce health disparities and bolster Thailand’s response to HIV and STI challenges.

## Introduction

HIV and sexually transmitted infections (STIs) remain a significant public health challenge globally, including in Thailand [1]. While Thailand has seen a 50% decrease in new HIV infections since 2010, vulnerable populations like transgender individuals, youth, sex workers, and men who have sex with men continue to be disproportionately affected [2]. This necessitates innovative prevention and treatment approaches.

Digital health interventions, particularly online platforms, have demonstrated potential in enhancing HIV/STI prevention and treatment efforts [3, 4]. These interventions can improve HIV testing, linkage to care, and treatment adherence [5, 6] and are particularly effective in reaching underserved populations [7–9] and overcoming geographic barriers. In Thailand, research has consistently demonstrated the effectiveness of online platforms, particularly in promoting HIV self-testing, facilitating care linkage [10–12], and addressing gaps in the HIV care continuum among young MSM and transgender women [13]. Furthermore, technology-driven interventions have proven successful in reducing sexual risk behaviors within these populations, reinforcing their role in public health strategies [14–17].

In Thailand, only some health care facilities or clinics have websites with an online appointment request mechanism, however the majority of key population-led health services (KPLHS) do not have an online booking mechanism. Given the high mobile online penetration in Thailand and the need for health services tailored to key populations disproportionately affected by or at risk of HIV, we identified the opportunity to create a platform that not only provides education and awareness about HIV and PrEP but also enables users to request appointments based on their location. Love2Test.org (L2T), managed by Love Foundation, is Thailand’s first mobile-friendly online platform that provides HIV and sexual health related information and education and additionally it connects users to clinics that offer HIV/STI testing, PrEP (Pre-Exposure Prophylaxis), PEP (Post-Exposure Prophylaxis), treatment, consultations, and gender-affirming hormone services. Accessible 24/7 via website or mobile app, it supports 48 clinics and ensures confidentiality with encrypted data storage [18, 19]. Users receive automated reminders for their appointments, and the platform caters to all population groups [20, 21]. L2T partners with community organizations to provide outreach and support to high-risk and underrepresented groups. Notifications and a support hotline help maintain medication adherence and mental health support [22, 23]. Additionally, the platform provides offline assistance, including transportation support, laboratory testing, and medical referrals. L2T went live in December 2020, and since then, more than 500,000 website visits have been recorded, with numbers continuing to grow as it remains widely used across Thailand.

This study aimed to identify patterns and trends by analyzing online engagement data, including website visits and service bookings from L2T, exploring potential correlations between users’ demographic factors and requested services. Furthermore, we assessed the platform’s effectiveness in reaching diverse user groups and addressing their specific needs.

## Methods

### Data Collection

The dataset was obtained from L2T, which collected data from October 1, 2023, to September 30, 2024, on website visits and service bookings. After removing duplicates and automated agents, 158,639 website visits with timestamps were recorded. The primary dataset included 8,865 service bookings, each characterized by the *province* where the booking was made, the list of HIV-related *requested services*—such as HIV Testing, STI Screening, PrEP, HIV Treatment, PEP, and Hormone Testing—the *booking date*, the user’s *age* at the time of booking, and their *self-identified gender.*

### Data Analysis Methods

We employed various analytical methods, comprising descriptive and service utilization analyses of demographic booking data, along with clustering, to clarify user interactions on the L2T platform. These analyses yielded a thorough understanding of user behavior and service needs, guiding the development of tailored interventions to improve health outcomes and optimize resource allocation.

#### Descriptive Analysis

Descriptive analysis extracts fundamental statistics from website visit data and summarizes key features of the booking dataset by computing statistical values and providing a comprehensive overview of booking information.

#### Service Utilization Analysis

To evaluate platform service utilization [24], demographic data from service bookings and website visits were analyzed. The analysis focused on the demographic distribution of gender, age, and location in relation to service bookings. Key demographic trends were identified through frequency counts and proportions. Furthermore, an analysis of website visitation and bookings monthly revealed service usage trends. The outcomes aimed to elucidate usage patterns that could inform targeted health initiatives, optimize resource distribution, and enhance service strategies.

#### Clustering Analysis

We used K-means clustering to categorize individuals based on their service booking behavior, demographics, and service utilization. This method, widely used in healthcare, helps uncover complex patient profiles and hidden data patterns [25, 26]. The goal of this analysis was to identify distinct user segments with unique characteristics and needs, which could inform the development of targeted interventions and outreach strategies. Grouping individuals into similar clusters enhances the understanding of different L2T user types and their specific service requirements.

## Results

### Descriptive Analysis

**Fig. 1:**
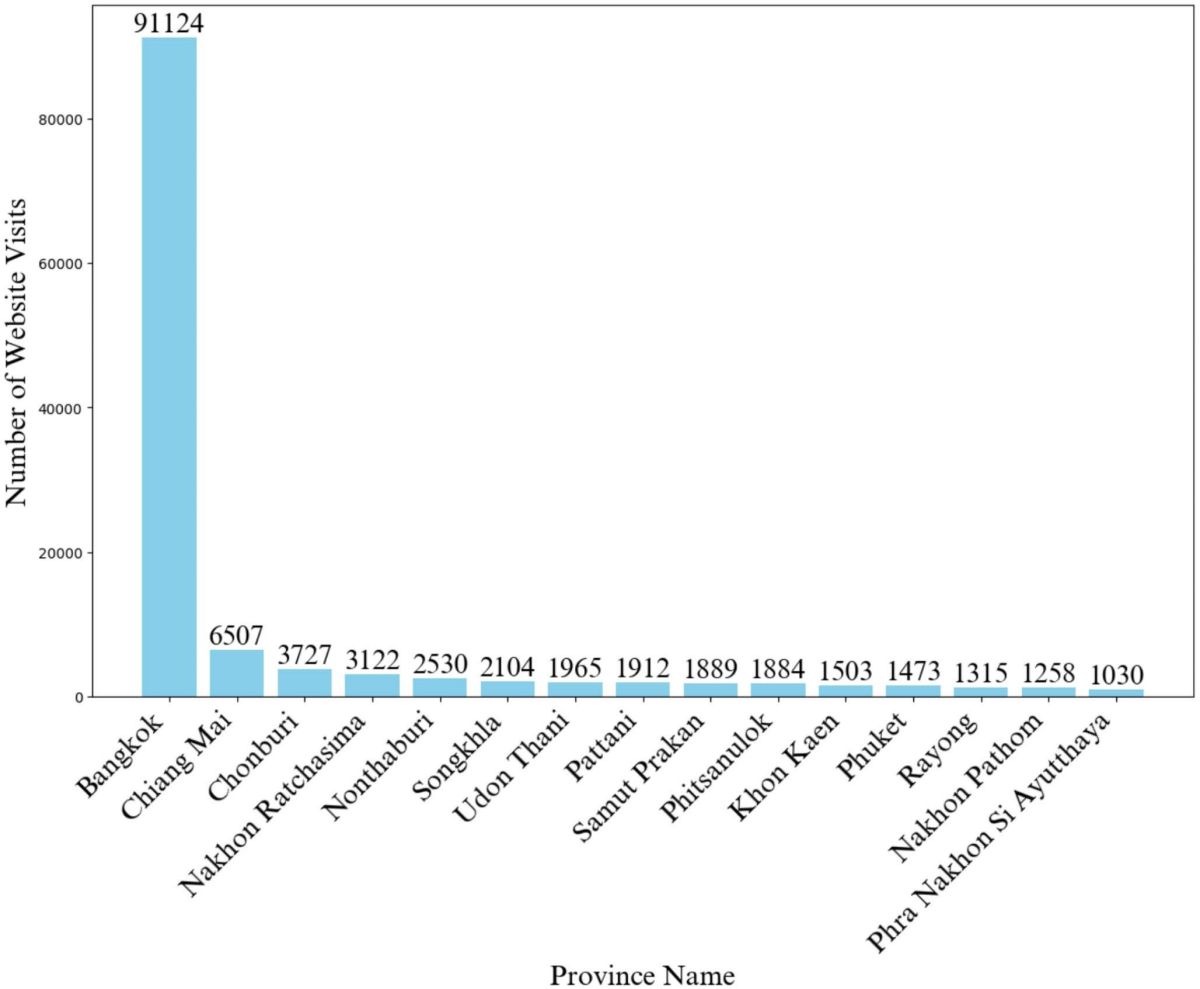
Top 15 provinces by website visits, highlighting the dominance of Bangkok

Between October 1, 2023, and September 30, 2024, L2T recorded 158,639 website visits, resulting in 8865 bookings—an overall visiting-to-booking rate of 5.59%. Of the total website visits, 134,423 originated from locations in Thailand, accounting for 84.73% of all website visits. Figure 1 highlights the top 15 provinces in Thailand ranked by website visits, with Bangkok receiving the highest number, followed by Chiang Mai, Chonburi, Nakhon Ratchasima, and Nonthaburi. Among the 8,865 bookings, 5,619 (63.38%) included multiple services, while 3,246 were for a single service, totaling 18,146 services, as shown in Table 1.

**Table 1:** Distribution of requested services by Province and service category

Region	Province	Total requested services	HIV testing	PrEP	STIs screening (excl HIV)	PEP	STIs treatment (excl HIV)	Hormone testing
Central	Bangkok	13,334	4,908	3,963	3,853	277	76	257
	Nakhon Pathom	188	81	26	44	30	0	7
	Total	13,522	4,989	3,989	3,897	307	76	264
Northern	Chiang Mai	431	199	86	115	16	15	0
	Chiang Rai	113	58	22	33	0	0	0
	Phayao	48	25	4	16	3	0	0
	Phitsanulok	138	60	34	44	0	0	0
	Nakhon Sawan	43	13	12	11	5	0	2
	Total	773	355	158	219	24	15	2
Northeastern	Khon Kaen	482	169	89	154	8	62	0
	Nakhon Ratchasima	260	116	71	73	0	0	0
	Ubon Ratchathani	461	166	83	123	63	0	26
	Udon Thani	137	59	22	42	14	0	0
	Total	1,340	510	265	392	85	62	26
Eastern	Chon Buri	1,517	600	361	405	0	0	151
	Rayong	128	68	13	39	0	0	8
	Total	1,645	668	374	444	0	0	159
Southern	Phuket	43	10	8	16	3	6	0
	Songkhla	808	367	172	112	115	0	42
	Surat Thani	15	4	6	3	2	0	0
	Total	866	381	186	131	120	6	42
Grand total	All Regions	18,146	6,903	4,972	5,083	536	159	493

The table presents the total number of requested services across different provinces

**Fig. 2:**
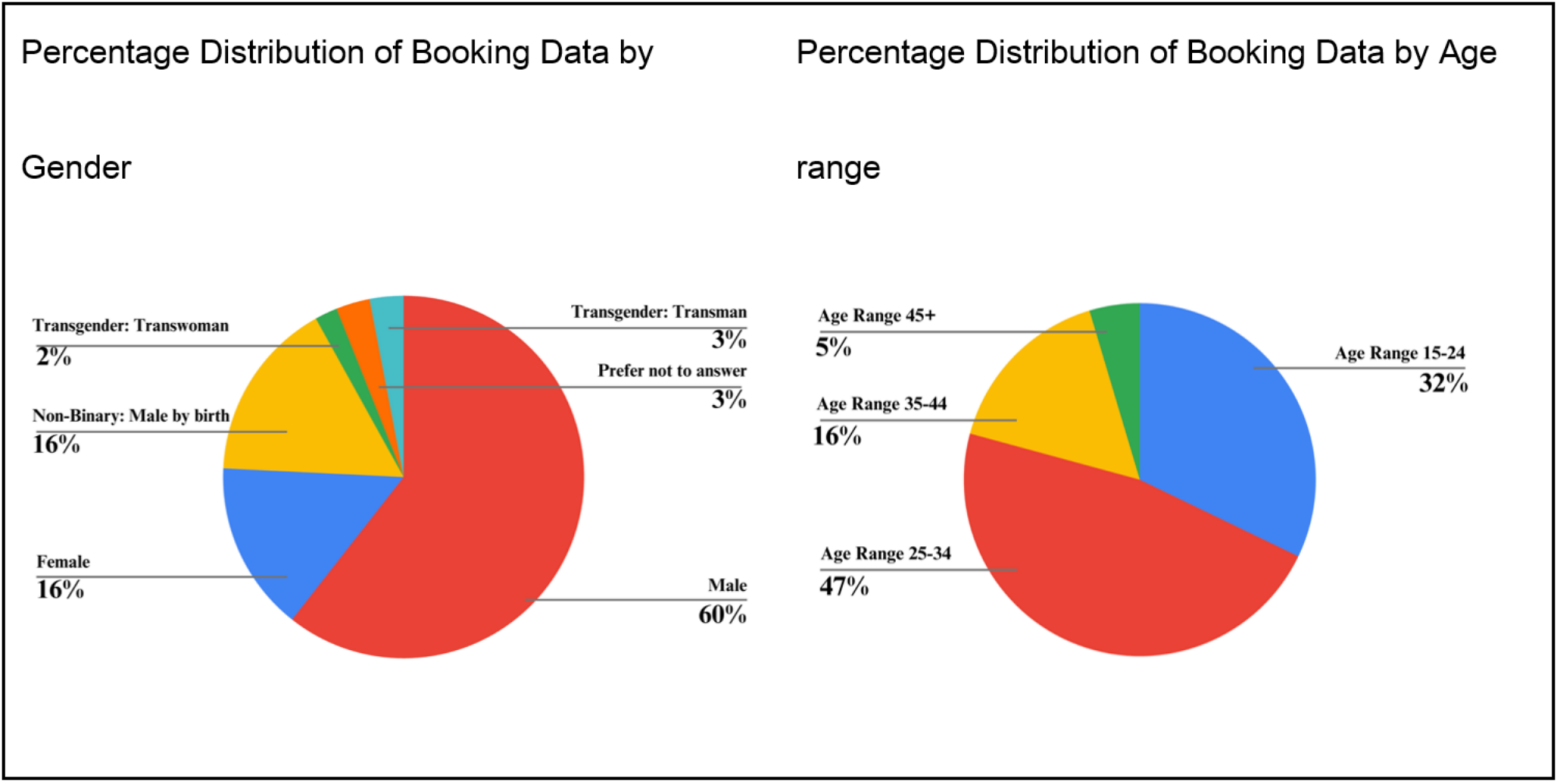
Percentage distribution of booking data by gender (left) and age range (right). The gender distribution shows that 60% of bookings were made by male users, followed by 16% female and 16% non-binary (male by birth). The age distribution indicates that the majority of bookings (47%) were made by individuals aged 25–34, followed by 32% from the 15–24 age group

The average age of individuals who booked appointments was 29.0 years (SD = 7.7, range 15–71, median 27). As illustrated in Fig. 2, the majority of bookings were made by individuals identifying as male (59.6%), followed by 16.4% identifying as non-binary, male at birth.

### Service Utilization Analysis

We analyzed service utilization by integrating demographic factors such as geographic location, age, and self-identified gender. Furthermore, we assessed service utilization based on booking times and website visiting times, enabling the exploration of usage patterns and trends across different months.

#### Service Utilization Analysis—by Demographic

**Fig. 3:**
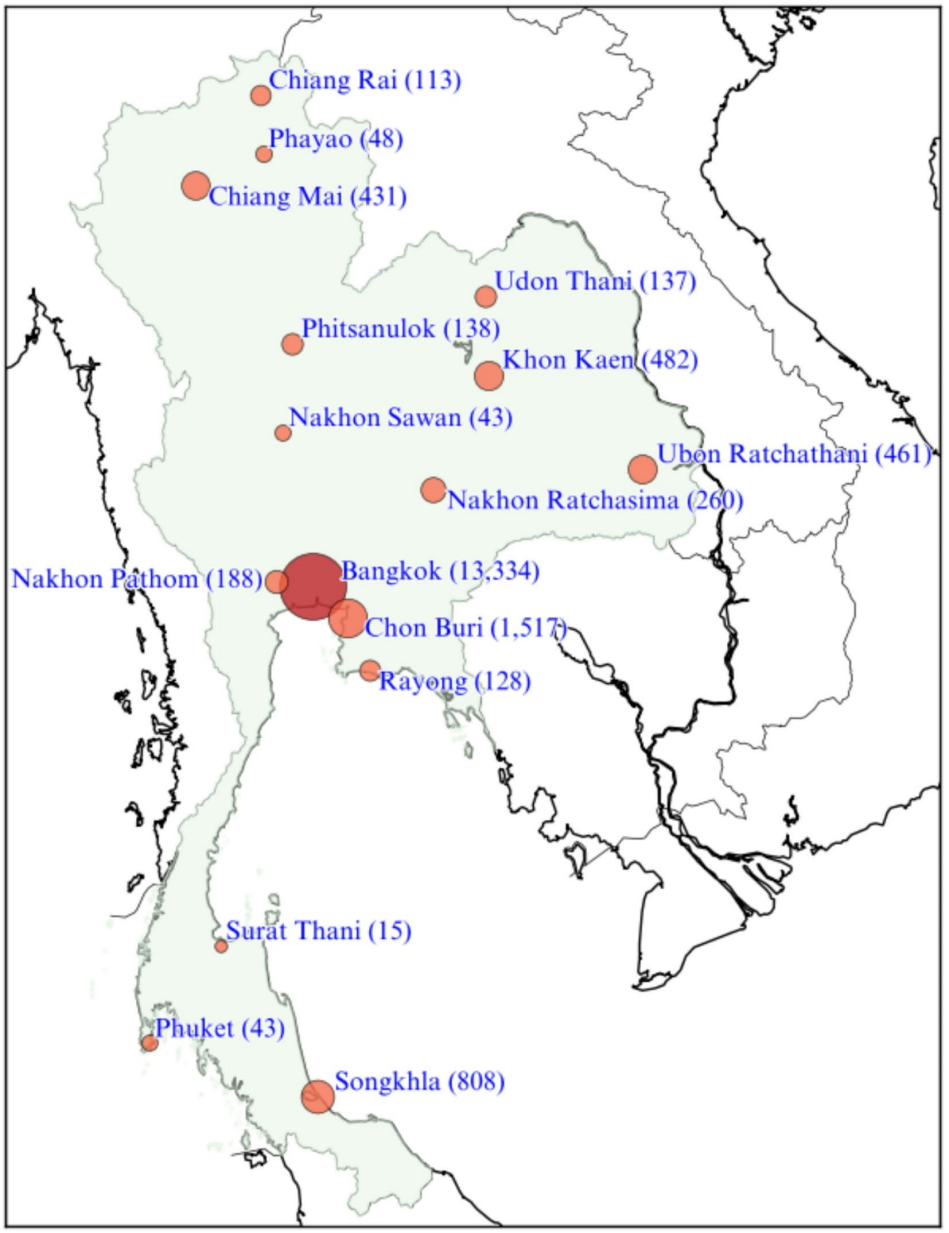
Geographical distribution of requested services across major cities in Thailand. The size of the circles represents the volume of requested services in each location, with Bangkok having the highest number (13,334), followed by Chon Buri (1,517) and Songkhla (808). Other notable cities with significant bookings include Khon Kaen (482), Ubon Ratchathani (461), and Chiang Mai (431)

Figure 3 illustrates the L2T platform’s broad reach across Thailand, with the highest engagement observed in Bangkok, which accounted for 13,334 services. Chon Buri (1,517 services) and Songkhla (808 services) ranked as the second and third most engaged locations, respectively. Notably, the platform also showed strong engagement in upcountry Thailand, with Khon Kaen (482 services), Ubon Ratchathani (461 services), and Chiang Mai (431 services) among the top locations outside the central region.

Table 1 provides detailed service numbers that elucidate the specific usage patterns across provinces. According to Table 1, HIV testing and STI screening were the predominant services requested, with 6,903 and 5,083 bookings, respectively. This reflects a significant demand for HIV and STI services among L2T users. Furthermore, the considerable number of PrEP service bookings (4,972) indicates heightened awareness and interest in PrEP among users. Conversely, hormone testing and PEP services exhibited diminished demand, with only 493 and 536 bookings, respectively. Bangkok dominates in HIV testing and PrEP service use, contributing 4,908 (27%) and 3,963 (22%) of national totals, respectively. Chon Buri ranks second with 600 HIV tests and 361 PrEP requests, signifying 3.3% and 2.0% of overall services. In contrast, Chiang Mai, the fifth most populated city of Thailand, exhibited minimal engagement with 199 HIV tests and 86 PrEP bookings, constituting 1.1% and 0.5% of total requested services. Phuket, a well-known hub for tourism and nightlife, reported among the three lowest figures, with a total of only 43 requested services, including just 10 HIV tests and 8 PrEP requested services.

Table 2 illustrates the allocation of healthcare service appointments by age with the age group 25–34 having the most requested services at 8,670 total requested services, followed by the age group 15–24, with 5,901. Across all age groups, HIV testing had the most requests, followed by STI screening and PrEP appointments. Of note, the age group 25–34 had the most requests for PEP services, with 218 total requested services (refer to Table 3 in the appendix for further details).

**Table 2:** Distribution of the number of requested services across age ranges

Age range	Total requested services	HIV testing	PrEP	STIs screening (excl. HIV)	PEP	STIs treatment (excl. HIV)	Hormone
15–24	5,901	2,310	1,459	1,670	206	67	189
25–34	8,670	3,275	2,419	2,472	218	68	218
35–44	2,802	1,024	877	728	85	20	68
45+	773	294	217	213	27	4	18
Grand total	18,146	6,903	4,972	5,083	536	159	493

The table presents the total number of requested services categorized by age group, covering available HIV-related services

#### Service Utilization Analysis—by Visiting and Booking time

**Fig. 4:**
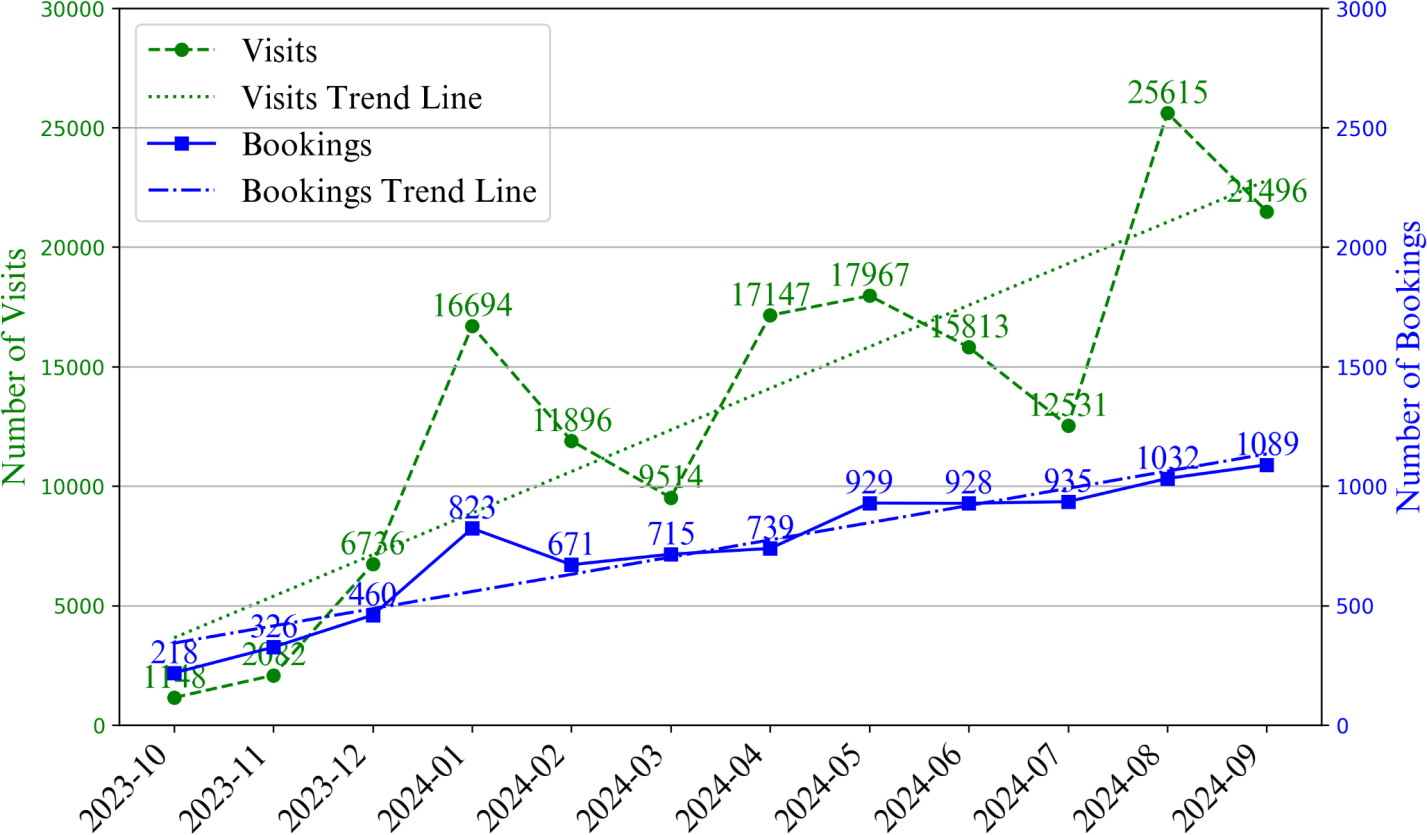
Monthly trends in visits and bookings on the L2T platform (October 2023–September 2024). The graph illustrates the number of visits and the number of bookings over time. Both visits and bookings show an overall upward trend, with significant peaks in January 2023, and August 2024, where visits reached their highest point at 25,615

The comparison of user activity on the L2T platform in Fig. 4 highlights fluctuating but generally upward trends in visits and bookings across different months. The figure shows three sharp increases in website visits: from December 2023 to January 2024 (6,676 to 16,694), March 2024 to April 2024 (9,514 to 17,147), and July 2024 to August 2024 (12,531 to 25,615). Bookings, represented by the solid line with square markers in Fig. 4, show a consistent rise, peaking at 1,089 in September 2024, indicating steady user visiting-to-booking rate over time.

### Clustering Analysis

We further analyzed potential correlations between user characteristics, booking patterns, emerging trends, and user behavior using cluster analysis. K-means clustering identified three user segments on the L2T platform based on booking patterns, demographics, and service utilization, with the number of clusters determined using the Elbow Method and Silhouette Analysis. The three clusters include the following:

Cluster 1 primarily consists of older male individuals utilizing HIV testing services.

Cluster 2 is composed of a mixture of younger and middle-aged individuals, primarily utilizing PrEP and STI screening services.

Cluster 3 is composed mainly of younger male and non-binary individuals who predominantly access HIV testing and STI screening services yet exhibit low PrEP utilization.

## Discussion

L2T was developed with the intention to provide a safe place for key and vulnerable populations to access HIV resources and information and additionally have the capability to connect individuals to clinics and services through an online appointment and consultation mechanism.

Between October 1, 2023, and September 30, 2024, L2T was most effective in reaching adults, aged 25 and older, which accounts for 68% of all bookings and 60% of users were identified as male. According to the UNAIDS 2023 data, in Thailand, youth accounts for about 50% of new HIV infections and only 45% of them were aware of PrEP. In our review, the age group 15–24 had the second most bookings for all HIV related services; this suggests that L2T was effective in reaching this vulnerable population. However, more work needs to be done to better reach this population including targeted awareness campaigns, using influencers, and peer to peer networking.

UNAIDS 2023 data for Thailand [27], highlighted that transgender individuals had an HIV prevalence of 2.2%, which was much higher than the general population prevalence of 1.1%. This underscored the importance of prioritizing support for this marginalized population. Notably, transgender individuals had the lowest engagement on the L2T platform across all age groups, likely due to a lack of clinics where the transgender community feels safe and supported. To address this gap, it is essential to expand services that offer trans-specific resources and comprehensive, wrap-around healthcare services, including HIV prevention and treatment as well as mental health support.

The majority of L2T users resided in the Bangkok metropolitan area, accounting for 74% of all bookings. Chiang Mai, Thailand’s fifth most populous province and a world-famous travel hotspot, is renowned for its cultural heritage, stunning architecture, and vibrant history. It was once ranked as the world’s top destination for digital nomads–remote workers who travel while working online–due to its low cost of living, coworking spaces, and reliable high-speed internet [28]. Nevertheless, despite these advantages, online visitor engagement and service bookings in Chiang Mai remained considerably lower than in Bangkok. Similarly, major cities such as Khon Kaen and Chon Buri also showed low engagement, despite their sizable populations and urban infrastructure. Likewise, Phuket, one of Thailand’s most popular tourist destinations, exhibited low activity. Several factors contribute to the discrepancies in service utilization across provinces. The primary reason for this disparity is the concentration of awareness campaigns, promotional activities, and collaborations with local community organizations in Bangkok. Most community organizations are based in the capital, giving L2T greater reach compared to other provinces. Additionally, the availability of KPLHS facilities is limited outside Bangkok, reducing opportunities for visitors in these areas to schedule appointments. Lastly, many individuals in other regions rely on local clinics and hospitals for sexual health services, including PrEP, which typically operate their own booking systems, further limiting engagement with L2T. To bridge this gap, efforts should focus on expanding Bangkok’s successful strategies—such as community partnerships, targeted outreach, and increased KPLHS facility access—to other provinces. Strengthening awareness campaigns and integrating L2T into existing regional healthcare networks will help ensure more equitable access to services across Thailand. Despite ranking among the top 15 in website visits, provinces such as Nonthaburi, Pattani, Samut Prakan, and Phra Nakhon Si Ayutthaya currently lack available clinics or booking services, highlighting a strong demand for expansion in these areas. To bridge this gap and better serve users, we recommend expanding clinic availability in these high-traffic locations.

Based on trends in visits and bookings (see Fig. 4), L2T has demonstrated consistent growth. Spikes in visits occurred in January, April, and August 2024, with a modest rise from April to May, followed by a decline from May to July before climbing again, each surge likely influenced by external factors. The January peak potentially resulted from New Year health commitments or marketing efforts by the dating application Hornet. The rise in April coincided with Thailand’s Songkran Festival, a nationwide celebration that may have influenced user engagement. The continued increase from April to May was likely driven by website activity related to the Mr. Gay HIV testing campaign promotion event in May. After this period, visits appeared to return to typical levels. In August, the surge in visits was likely due to promotional initiatives by organizations such as TestBKK.org, which launched that month to support Bangkok’s LGBTQI + community through HIV testing and advocacy. While visit trends showed sharper fluctuations, particularly in August, service bookings grew at a steadier pace. This suggests that users often took time to transition from browsing the platform to making a booking, especially following periods of high traffic. Overall, the upward trend in both visits and bookings indicate increasing awareness and trust in L2T, with visits experiencing periodic surges and bookings gradually rising over time.

The cluster analysis identified three distinct user segments. While the L2T platform offers PrEP awareness and resources, only Cluster 2 actively booked PrEP services, whereas Clusters 1 and 3 primarily used HIV testing and STI screening services. Cluster 1, which consisted of older males, was less likely to engage in high-risk sexual behavior or rely on other forms of HIV prevention, such as condoms. However, they could still benefit from HIV prevention education and increased awareness of PrEP. Cluster 3, composed of younger individuals, showed a concerning trend. Despite their higher risk for HIV acquisition, based on their age and requested services, there was little engagement with PrEP services. This is alarming, as it indicates that our PrEP awareness campaigns have not been effectively reaching this high-risk group. To better serve these individuals, it is crucial to develop tailored interventions that address their unique needs. This includes enhancing PrEP awareness campaigns to be more impactful and expanding access to PrEP services through tele-PrEP, mobile clinics, and extended office hours. By leveraging these insights, L2T can implement more effective outreach strategies, ultimately improving the effectiveness of health campaigns and support services.

In our analysis, we noted a significant visitor volume; however, the visiting-to-booking rate was merely 5.59%. This proposed service enhancements and outreach initiatives may help increase the platform’s visiting-to-booking rate.

## Limitation

This study has several limitations. As an observational, retrospective analysis, it is subject to the inherent constraints of retrospective data, including the absence of predefined parameters. The website visit data were high-level, capturing metrics such as visit count, IP address, general geographic location, and timestamp. However, they lacked detailed demographic and health information, such as HIV status, age, or other relevant characteristics. Similarly, the bookings data did not include reports on no-shows, test results, prescription details, or barriers to accessing services, as these were not provided by clinics. This limits our ability to assess service outcomes. Additionally, L2T offers service bookings at only 48 clinics, primarily in Bangkok, with the remainder located in other major cities. This geographical concentration further restricts the generalizability of our findings.

## Conclusion

L2T serves as a vital bridge to care for vulnerable and marginalized key populations in Thailand. By offering a confidential and accessible platform, L2T helps mitigate the impact of stigma and discrimination, enabling individuals to seek HIV and sexual health information with privacy and dignity. It has expanded access to care, particularly for older adults and young people. However, challenges remain in engaging transgender individuals, extending outreach beyond Bangkok, and increasing PrEP awareness among high-risk youth. Since its inception, L2T has accumulated extensive data that can be analyzed to generate valuable insights. These findings can be shared with the medical community, researchers, and policymakers to inform strategic decisions, optimize service delivery, and shape public health policies that enhance care and support for those most in need.

## Data Availability

This study utilized L2T platform data from the Love2Test.org, including website visit and booking data collected over one year (October 1, 2023– September 30, 2024). Access to the data is available upon request and subject to approval. For further details, please contact the corresponding author. The coded implementation is publicly available at https://github.com/wtepsan/Analysis-of-HIV-Related-Services-in-a-Major-Thai-City.

## Appendix

**Table 3:** Distribution of requested services by age range and self-identified gender

Age range	Self-identified gender	Requested services	HIV testing	PrEP	STIs screening	PEP	STIs treatment	Hormone testing
15–24	Male	3,068	1,215	807	856	100	39	51
	Female	1,094	449	177	369	31	18	50
	Transgender: transman	197	73	42	51	13	1	17
	Transgender: transwoman	162	49	26	35	4	1	47
	Non-binary: male by birth	1,138	428	346	294	50	6	14
	Non-binary: female by birth	21	9	2	7	3	0	0
	Prefer not to answer	221	87	59	58	5	2	10
	Total	5,901	2,310	1,459	1,670	206	67	189
25–34	Male	5,276	1,968	1,546	1,505	132	46	79
	Female	1,180	516	197	365	29	8	65
	Transgender: transman	266	102	72	73	6	1	12
	Transgender: transwoman	201	62	53	46	5	1	34
	Non-binary: male by birth	1,470	526	466	402	41	12	23
	Non-binary: female by birth	11	5	2	3	0	0	1
	Prefer not to answer	266	96	83	78	5	0	4
	Total	8,670	3,275	2,419	2,472	218	68	218
35–44	Male	1934	688	642	504	48	15	37
	Female	321	144	64	88	15	1	9
	Transgender: transman	73	26	22	15	4	0	6
	Transgender: transwoman	39	12	11	7	1	0	8
	Non-binary: male by birth	352	125	112	92	13	4	6
	Non-binary: female by birth	0	0	0	0	0	0	0
	Prefer not to answer	83	29	26	22	4	0	2
	Total	2,802	1,024	877	728	85	20	68
45+	Male	575	211	166	162	22	4	10
	Female	65	35	13	14	0	0	3
	Transgender: transman	16	7	2	6	0	0	1
	Transgender: transwoman	3	1	0	1	0	0	1
	Non-binary: male by birth	99	34	33	26	4	0	2
	Non-binary: female by birth	3	1	0	1	0	0	1
	Prefer not to answer	12	5	3	3	1	0	0
	Total	773	294	217	213	27	4	18
Grand total		18,146	6,903	4,972	5,083	536	159	493

Building upon the information in Tables 2 and 3 provides a more detailed breakdown of healthcare requested services by age group and self-identified gender. The 25–34 age group leads with 8,670 requested services, followed by the 15–24 age group with 5,901. HIV testing was the most requested service, followed by STI screening and PrEP. Notably, the 25–34 age group also had the highest demand for PEP services, totaling 218 requests.

HIV Testing is the most booked service across all genders and age groups, followed by STI Screening and PrEP. The 25–34 age group had the highest demand for PEP (218 bookings). Hormone Testing is primarily utilized by Transgender and Non-Binary individuals, with significant bookings in the younger age groups.

The data reveals elevated health service utilization among young adults, predominantly males. Transgender and Non-Binary individuals exhibit lower healthcare access, indicating a necessity for enhanced accessibility. The diminished utilization in the 45 + demographic implies possible obstacles or changing health priorities. This analysis underscores the critical need for healthcare accessibility across all gender identities, especially for transgender and non-binary individuals.
